# Structure-to-function relationships of bacterial translocator protein (TSPO): a focus on *Pseudomonas*

**DOI:** 10.3389/fmicb.2014.00631

**Published:** 2014-11-19

**Authors:** Charlène Leneveu-Jenvrin, Nathalie Connil, Emeline Bouffartigues, Vassilios Papadopoulos, Marc G. J. Feuilloley, Sylvie Chevalier

**Affiliations:** ^1^Laboratory of Microbiology Signals and Microenvironment EA 4312, University of RouenEvreux, France; ^2^Department of Medicine, Research Institute of the McGill University Health Centre, McGill UniversityMontreal, QC, Canada

**Keywords:** translocator protein, TSPO, bacteria, *Pseudomonas*, structure, function

## Abstract

The translocator protein (TSPO), which was previously designated as the peripheral-type benzodiazepine receptor, is a 3.5 billion year-old evolutionarily conserved protein expressed by most Eukarya, Archae and Bacteria, but its organization and functions differ remarkably. By taking advantage of the genomic data available on TSPO, we focused on bacterial TSPO and attempted to define functions of TSPO in *Pseudomonas* via *in silico* approaches. A *tspo* ortholog has been identified in several fluorescent *Pseudomonas*. This protein presents putative binding motifs for cholesterol and PK 11195, which is a specific drug ligand of mitochondrial TSPO. While it is a common surface distribution, the sense of insertion and membrane localization differ between α- and γ-proteobacteria. Experimental published data and STRING analysis of common TSPO partners in fluorescent *Pseudomonas* indicate a potential role of TSPO in the oxidative stress response, iron homeostasis and virulence expression. In these bacteria, TSPO could also take part in signal transduction and in the preservation of membrane integrity.

## INTRODUCTION

**FIGURE 1:**
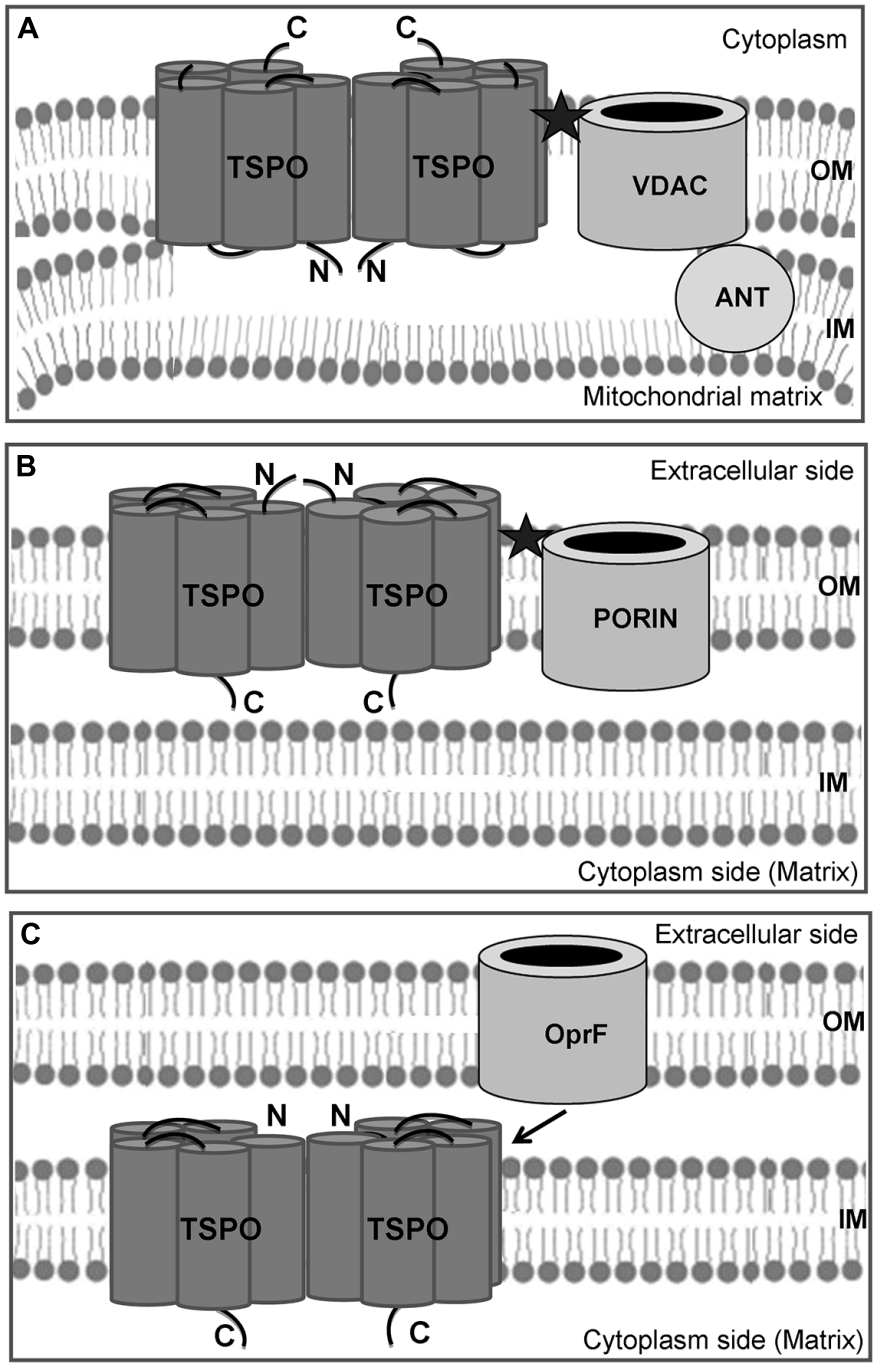
**Schematic cellular localization of TSPO.** The mitochondrial TSPO is located at the level of the outer–inner mitochondrial membrane contact sites, and it forms a ternary association with the voltage-dependent anionic channel (VDAC) and the adenine nucleotide transport protein (ANT; **A**). In *Rhodobacter sphaeroides* 2.4.1, TSPO is also located in the outer membrane, but it is oriented in opposite sense with the C-terminal end facing the cytoplasmic side **(B)**. In *Pseudomonas fluorescens*, TSPO is oriented in the same sense as in *R. sphaeroides*, but it appears to be located in the inner membrane **(C)**. TSPO has functional relationships with the major outer membrane porin OprF, but the two proteins cannot associate in the same membrane and form a benzodiazepine-binding site (★), as observed in mitochondria and *R. sphaeroides*.

The translocator protein (TSPO), which was initially designated as the peripheral-type benzodiazepine receptor (PBR), was discovered as a diazepam-binding site in kidney (Braestrup and Squires, 1977), and found to be abundant in the outer mitochondrial membrane of steroid-synthesizing cells, including those in the central and peripheral nervous systems (Papadopoulos et al., 2006). Mitochondrial TSPO is associated to the voltage-dependent anionic channel (VDAC; also designated as mitochondrial porin) and to the adenine nucleotide transport protein adenine nucleotide translocase (ANT; McEnery, 1992; Papadopoulos et al., 2006, 2007) or the ATPase family AAA domain-containing 3A protein (Rone et al., 2012), forming a transmembrane hyperstructure (**Figure 1A**). TSPO has been involved in many physiological functions in mammals, including cell growth and proliferation, immunomodulation, mitochondrial respiration, apoptosis, and adaptation to oxidative stress (Wang et al., 1984; Ruff et al., 1985; Hirsch et al., 1989; Papadopoulos et al., 2006). One of its main functions is its implication in cholesterol import to the inner mitochondrial membrane, the rate-limiting step in steroid hormone biosynthesis (Papadopoulos et al., 1997a). TSPO has been shown to be a high affinity cholesterol-binding protein (Jamin et al., 2005), and to function together with other proteins in a complementary manner and within a large protein complex, to mediate the import of cholesterol into mitochondria (Miller and Bose, 2011; Issop et al., 2013; Poderoso et al., 2013). The function of TSPO as translocator or as acceptor of molecules (including cholesterol) associated with larger membrane translocator complexes is yet still unclear. TSPO drug ligands were shown to significantly affect cell and tissue steroid production and regulate circulating and tissue steroid levels (Krueger and Papadopoulos, 1990; Papadopoulos et al., 1990, 1997b, 2006; Lacapère and Papadopoulos, 2003; Levine et al., 2007; Veenman et al., 2007; Rupprecht et al., 2009, 2010; Chung et al., 2013), indicating that TSPO is required for steroidogenesis. However, recent studies using a Leydig- and Sertoli-cell targeted knock out of the *tspo* gene (Morohaku et al., 2014) and a *tspo* null mice (Tu et al., 2014) indicated that the presence of TSPO may not be essential for steroid hormone biosynthesis. Although this raised controversy and several questions regarding mitochondrial TSPO function in steroidogenesis (Papadopoulos, 2014), and essential cell life processes, this does not detract from the fact that TSPO is abundant in steroidogenic cell mitochondria and that drug ligands act specifically on this protein to increase cholesterol import into mitochondria and steroidogenesis (Papadopoulos et al., 1997b, 2006; Lacapère and Papadopoulos, 2003; Levine et al., 2007; Veenman et al., 2007; Rupprecht et al., 2009). Recent structural studies confirmed the proposed structure and function of TSPO drug ligands (Jaremko et al., 2014). TSPO upregulation has been connected to several diseases, including cancer (Batarseh and Papadopoulos, 2010), neuronal damage, neurodegeneration, and inflammation, making the protein an important marker for glial cell activation and neuroinflammation (Harberts et al., 2013; Dickens et al., 2014). Recently, TSPO has attracted attention as a possible molecular target for tumor imaging and chemotherapy (Austin et al., 2013), and initial clinical trials have indicated that TSPO ligands might be valuable in the treatment of neurological and psychiatric disorders (Rupprecht et al., 2010).

Considering these important functions and perspectives in mammals, TSPO has been the focus of multiple studies and reviews (Papadopoulos et al., 2006; Rupprecht et al., 2010; Fan et al., 2012). However, it remains a mysterious protein, since functional links between its putative translocation activity and its implication in multiple physiological functions are still lacking (Fan et al., 2012). Since TSPO was conserved throughout evolution in among the great diversity of Eukarya, Archaea, and Bacteria (Chapalain et al., 2009; Fan et al., 2012), and since bacterial and mammalian TSPOs are functionally interchangeable (Yeliseev et al., 1997), it is tempting to assume that the more ancestral cell forms – the bacteria – could provide further insights into the TSPO structure-to-activity relationships. The present study aims to describe bacterial TSPO in terms of its structure and function on the basis of published data and *in silico* predictive approaches, with a special focus on TSPO belonging to the highly adaptable *Pseudomonas* genus.

## BACTERIAL TSPO

### STRUCTURE

The first bacterial protein homolog of PBR was described in the non-sulfurous photosynthetic purple bacterium *Rhodobacter sphaeroides* by Yeliseev and Kaplan (1995). In that paper, the nomenclature tryptophan-rich sensory protein (TSPO) was proposed. The 17 kDa bacterial protein is composed of 158 amino acids and shows only 33.5 and 21% of similarity and identity respectively with human TSPO (Yeliseev et al., 1997). Thanks to molecular modeling, *R. sphaeroides* TSPO was proposed to fold into the outer membrane (OM) as five hydrophobic α helix regions, similarly to eukaryotic TSPO (Yeliseev and Kaplan, 2000), suggesting that the protein was conserved at the topological rather than at the amino acid sequence level. The postulated functional form of *R. sphaeroides* TSPO is a homodimer (Yeliseev and Kaplan, 2000). The three-dimensional structure of mammalian TSPO in complex with the PK 11195 drug ligand was recently reported and confirmed the previously reported TSPO topography, drug and cholesterol-binding sites as well as provided a model for ligand induced cholesterol transport (Jaremko et al., 2014). However, as shown in **Figures 1A,B**, the *R. sphaeroides* and mitochondrial TSPOs are proposed to be inserted in opposite directions, i.e., the N-terminus is exposed to the extracellular side of the bacterial protein (Yeliseev and Kaplan, 2000), while it is located at the interface of the inner and outer mitochondrial membranes. One hypothesis that may account for this particularity is that while in bacteria, the protein is produced in the cytoplasm and then addressed into the OM from the inside, in eukaryotes the *tspo* gene has been translocated to the nuclear DNA (Gray et al., 2001) probably a billion years ago; therefore, TSPO is synthesized in the cell’s cytoplasm and inserted in the mitochondrial membrane from the outside via translocase Tom70 (Otera et al., 2007) and metaxin 1 (Rone et al., 2009). The localization of TSPO in the OM of *R. sphaeroides* is further consistent with the observation that benzodiazepines such as flunitrazepam can crosslink TSPO to its major OM porin, as occurs in mammals (Yeliseev and Kaplan, 1995). This association may indeed improve the recognition of benzodiazepines in mammals, since the binding site for these artificial ligands could be located in the cleft between the two proteins (**Figure 1**, black star; McEnery, 1992; Lacapère et al., 2001; Veenman et al., 2007), albeit TSPO alone could be involved in the mitochondrial protoporphyrin IX (PPIX) import (Wendler et al., 2003).

### FUNCTIONS

As in Eukarya, the postulated roles of TSPO in bacteria are numerous, but we can begin to identify their common general functions. The first periplasmic loop of *R. sphaeroides* TSPO contains a high percentage (22%) of tryptophan residues, reflecting possibly a WWD heme-binding domain that is typical of proteins involved in heme membrane transport (Goldman et al., 1998). Mitochondrial TSPO was previously shown to bind PPIX (Kinnally et al., 1993; Taketani et al., 1995), suggesting that TSPO may have a role in mitochondrial processing of PPIX via the heme synthesis pathway (Furre et al., 2005; Veenman et al., 2007). In *R. sphaeroides*, TSPO appears to be involved in controlling the eﬄux of tetrapyrrole intermediates of the heme/bacteriochlorophyll biosynthetic pathway, and it could act as a negative regulator of photosynthetic gene expression and pigment synthesis in response to variations of oxygen and/or light availability (Yeliseev and Kaplan, 1995). The mechanism by which TSPO in *Rhodobacter* controls the transport of porphyrins remains to be determined. Interestingly, in the plant *Arabidopsis thaliana* (At), AtTSPO has been proposed to participate in the interactions between the plastidal and the mitochondrial tetrapyrrole biosynthetic pathway (Lindemann et al., 2004). This complex function of TSPO in the response to environmental conditions was confirmed in *R. capsulatus*, where TSPO is highly expressed in anaerobic conditions and in the absence of light (Bauer, 2004). TSPO indeed down-regulates transcription of bacterio-chlorophyll and carotenoid biosynthesis genes, as well as the *puc* operon, which encodes the structural proteins of the light-harvesting-II peripheral antenna complex (Yeliseev et al., 1997). The role of TSPO appears to be similar in cyanobacteria since it was shown to control photosynthetic genes expression in *Synechococcus* (González et al., 2011). Similarly, TSPO could be involved in the rapid shut down of the photosynthesis gene cluster in response to oxygen variations in the phototrophic bacterium, *Dinoroseobacter shibae* (Tomasch et al., 2011), and in the regulation of gene expression by light in the marine flavobacterium *Dokdonia* (González et al., 2011). More generally, TSPO has been proposed by Yeliseev et al. (1997) as an “oxygen sensor.” This protein could control porphyrin eﬄux by modulating the antirepressor/repressor AppA/PpsR system implicated in the regulation of photosynthetic gene expression in response to changes in oxygen partial pressure (Oh and Kaplan, 2001; Zeng and Kaplan, 2001). Noticeably, this activity is not specific to bacterial TSPO, since a mitochondrial *tspo* gene provided *in trans* in *R. sphaeroides* can replace the bacterial TSPO oxygen sensor function (Yeliseev et al., 1997). Interestingly, in the symbiotic *Sinorhizobium meliloti*, TSPO and FixL, an oxygen sensor, are involved in regulating the expression of the *ndi* locus that is specifically induced in nutrient (oxygen, carbon, nitrogen) deprivation conditions. TSPO appears to be epistatic to FixL, since no expression is observed in the *tspO* mutant, even though FixL is still present (Davey and de Bruijn, 2000). Further investigations are necessary to determine if these related TSPO homologs function in a similar manner and respond to the same signals. Nonetheless, these data suggest that TSPO is involved in regulating gene expression and is likely to provide a new and important way to think about signal transduction in prokaryotes.

## TSPO IN *Pseudomonas*

### GENOMIC ORGANIZATION, SEQUENCES, AND TOPOLOGY CONSERVATION

The structure–activity relationships of TSPO have been particularly investigated in *Pseudomonas fluorescens* MF37 (Chapalain et al., 2009). Here we have identified a *tspo* ortholog gene in 7 of the 48 fully sequenced genomes of *Pseudomonas* ^1^ (Winsor et al., 2011), among which are three *Pseudomonas syringae* (*pv. phaseolicola* 1448A, *pv. syringae* B728a, and *pv. tomato* DC3000), three *Pseudomonas fluorescens* (strains SBW25, Pfl0-1, A506), and one *Pseudomonas poae* (strain RE*1-1-14). *In silico* analysis will thus be focused on these strains. The genomic environment of *tspo* is not conserved among these seven strains, as previously described (Chapalain et al., 2009), although some degree of conservation exists in each species. For example, in two of the three studied *Pseudomonas fluorescens* strains, SBW25 and A506, *tspo* forms an operonic structure with an esterase-encoding gene that is involved in lipid metabolism^1^ (Winsor et al., 2011). Many transposase-encoding genes were found in the vicinity of *tspo* in the three *Pseudomonas syringae* strains. Transposases are usually included in autonomous mobile genetic elements, such as transposons or insertion sequences, and they are required for excising and inserting the mobile element, suggesting that in these three strains, *tspo* acquisition may be the result of an ancient horizontal transfer. Moreover, TSPO could be functionally related to PSPPH_2782 (a transposase of *Pseudomonas syringae pv. phaseolicola* 1448A ^2^), but the guanine–cytosine (GC) ratio of *Pseudomonas syringae tspo* is the same as in the rest of its genomic environment, suggesting that if *tspo* was acquired by insertion, the event was ancient.

**FIGURE 2:**
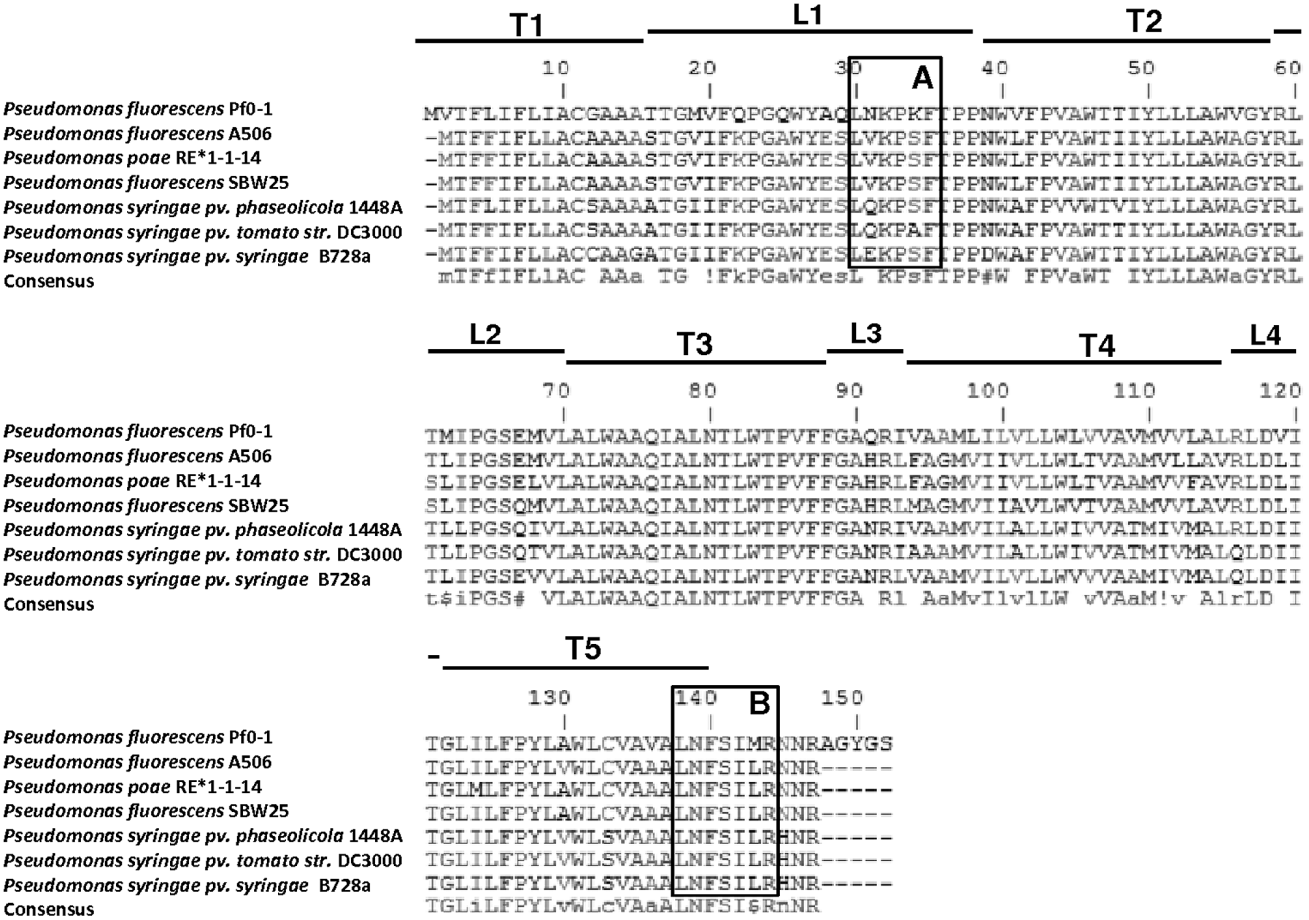
Amino acid sequence alignment of TSPO from sequenced *Pseudomonas* genomes, including *Pseudomonas fluorescens* (strains Pf0-1, SBW25, A506), *Pseudomonas poae* (strain RE*1-1-14), and *Pseudomonas syringae* (strains 1448A, DC3000, B728a). Framed are A) the PK 11195 binding site **LxKPsW/F** (Fan et al., 2012), and (B) the CRAC motif **L/V/I-(X)1-5-Y-(X)1-5-R/K** (Fan et al., 2012). T, transmembrane segment; L, Loop.

Base-pairing comparisons using the MultAlin algorithm ^3^ showed that TSPO was highly conserved among these seven strains at both the nucleotide and amino acid sequence levels, with similarities ranging from 71.6–91.1 and 76–95.9%, respectively. Protein secondary motifs were also conserved in *Pseudomonas* since a TopPred analysis ^4^ revealed that TSPO folds into five putative transmembrane α-helixes (**Figure 2**: T1–T5), similarly to *R. sphaeroides* and mitochondrial TSPO (Chapalain et al., 2009).

However, *Pseudomonas* TSPO was predicted to fold into the inner membrane (**Figure 1C**; Chapalain et al., 2009), whereas *R. sphaeroides* TSPO was predicted to insert in the OM as mitochondrial TSPO (**Figure 1B**, Yeliseev and Kaplan, 2000). This is consistent with the observation that in *Escherichia coli,* a recombinant eukaryotic protein was essentially retrieved from inner membrane extracts (Yeliseev et al., 1997), suggesting the different localization of TSPO in α- and γ-proteobacteria (Chapalain et al., 2009). Furthermore, the orientation of this insertion was in the opposite direction with respect to *Pseudomonas* and *R. sphaeroides* TSPO: in the case of *Pseudomonas* TSPO, the N-terminus was inserted into the inner membrane; conversely, it is exposed at the bacterial surface in *R. sphaeroides* (**Figures 1B,C**). Interestingly, in mammals, TSPO was also found in the cytoplasmic membrane (Oke et al., 1992; Woods and Williams, 1996; Woods et al., 1996), suggesting that TSPO could exert functions at different levels, including on the cell surface or outside the cell.

In addition, the potential PK 11195 binding motif LxKPsW/F, previously determined by Fan et al. (2012), was identified in the first loop (**Figure 2**). This motif was fully conserved among the seven TSPO sequences of fluorescent *Pseudomonas*, suggesting it may play a function in *Pseudomonas* TSPO. This binding motif was mapped onto the recently reported 3D structure of mouse TSPO just outside of the PK 11195 binding pocket and it consists of 65 contacts between the ligand and TSPO involving the five transmembrane domains (Jaremko et al., 2014). PK 11195 is an isoquinoline carboxamide drug ligand of the mitochondrial TSPO, whose binding site is exclusively located on TSPO (Le Fur et al., 1983). The use of PK 11195 in *Pseudomonas fluorescens* MF37 allowed researchers to demonstrate that there was a functional association between TSPO and the major OM porin OprF since the biological activity of PK 11195 was totally abolished in an OprF deletion mutant (Chapalain et al., 2009). Moreover, the affinity of PK 11195 for *Pseudomonas fluorescens* MF37 TSPO was the same as for mitochondrial TSPO, indicating the preservation of common properties between the two proteins (Chapalain et al., 2009). However, in normal and low-oxygen conditions, PK 11195 was without effect on the growth kinetics of *Pseudomonas fluorescens* MF37 (Chapalain et al., 2009), suggesting that it should not act as an oxygen sensor as in *R. sphareoides* (Yeliseev et al., 1997). The localization of TSPO in the OM of *R. sphaeroides* is consistent with the observation that benzodiazepines such as flunitrazepam can crosslink TSPO to its major OM porin, as in mammals (Yeliseev and Kaplan, 1995). While in mitochondria TSPO and VDAC are in the same (outer) membrane where they can associate to form a diazepam-binding site (**Figures 1A,B**), in *Pseudomonas fluorescens*, TSPO and porins (such as OprF) should be inserted in the inner and OMs, respectively (**Figure 1C**). This can explain the absence of sensitivity of *Pseudomonas fluorescens* to diazepam (our unpublished data) since, if TSPO also interacts with porin(s), the structure of the complex should be different. The conservation of the PK 11195 binding motif in the bacterial TSPO sequences suggests that molecules mimicking the structure of PK 11195 might interact with TSPO. The association of bacterial TSPO with OprF in case of *Pseudomonas fluorescens* MF37, or the major OM protein in case of *R. sphaeroides* further suggest that TSPO might be the binding site for such molecules and that it is associated with larger membrane channels, relatively similarly to mitochondrial TSPO associated to VDAC.

Taken together, these data show that *Pseudomonas* TSPO shares common structural properties with *R. sphaeroides* and mitochondrial TSPOs. It should be noted that the most intriguing observation included the topological differences found among several of the proteins between eukaryotic and prokaryotic TSPOs; however, the most striking difference was seen between *R. sphaeroides* and *Pseudomonas* TSPOs, suggesting that they hold common yet specific functions.

### PUTATIVE FUNCTIONS

The functional role of TSPO in these bacteria appears to be at least as essential as in *R. sphaeroides*, since while in this species a knock-down of TSPO is possible (Yeliseev and Kaplan, 1995), any major change of TSPO expression (positively or negatively) in all investigated strains of *Pseudomonas fluorescens* is lethal (Chapalain et al., 2009).

#### TSPO and cholesterol/steroidogenesis

As described in the introduction section, one of the functions of mitochondrial TSPO is its involvement in cholesterol import into mitochondria, which is a prerequisite for steroidogenesis – a function which was recently questioned due to the absence of observed effects on steroidogenesis in a *tspo*-null mice mutant– (Morohaku et al., 2014; Tu et al., 2014). Although these findings argue that cholesterol import into mitochondria may occur in the absence of TSPO, they do not indicate that in normal cells this process is not mediated by TSPO, a protein abundant in the outer mitochondrial membrane. Noticeably, the potential cholesterol recognition amino-acid consensus (CRAC) L/V/I-(X)_1-5_-Y-(X)_1-5_-R/K, previously determined in mammals by Li and Papadopoulos (1998), and shown to be common among many proteins (Fantini and Barrantes, 2013), and its evolution reviewed by Fan et al. (2012), was identified in the C-terminal part of TSPO (**Figure 2**). The CRAC motif lacked the internal characteristic Y of eukaryotes (**Figure 2**). This feature, associated with the absence of cholesterol in bacterial membranes, prompted Fan et al. (2012) to suggest that this motif is rarely found in bacterial TSPO sequences. Notably, this sequence was highly conserved among the seven *Pseudomonas* TSPOs, suggesting that this C-terminal part has a special function in the TSPO role in these bacteria. Cholesterol has not yet been found to be produced in bacteria. However some steroids, among which cycloartenol, the first oxidosqualene cyclization product in plants (Bode et al., 2003), or lathosterol, a molecule closely structurally-related to cholesterol, have been shown to be produced by several *Myxobacteria* species as well as by *Methylococcus capsulatus* (Bird et al., 1971; Tippelt et al., 1998; Bode et al., 2003; Gawas et al., 2011). Moreover, a phylogenetic study of the sterol biosynthesis pathways reported that some other bacterial genomes may possess certain genes which products could be involved in sterol biosynthesis pathways. In particular, this study reported that Myxobacterium *Plesiocystis pacifica* harbors the largest reported set of eukaryotic sterol-synthesizing enzyme homologs (Desmond and Gribaldo, 2009), suggesting that this Myxobacterium could be at the origin of these eukaryotic enzymes (López-Garćia and Moreira, 1999). It is also likely that *Plesiocystis pacifica* has acquired its pathway for sterol synthesis via horizontal gene transfer from eukaryotes (Desmond and Gribaldo, 2009).

In the Myxobacteria *Nannocystis exedens,* almost all known intermediates and side-products of the cholesterol biosynthesis in eukaryotes can be found under different culture conditions (Bode et al., 2003). Although the authors clearly demonstrate that Myxobacteria can produce as much steroids as eukaryotic organisms, the function of these compounds remains a mystery. Due to their hydrophobicity, these molecules are most probably located in membranes, where they might have a function in controlling fluidity, in a manner similar to that described for cholesterol in eukaryotes (Ourisson and Nakatani, 1994). An additional function of the myxobacterial steroids might be to act as signaling molecules, similar to steroid hormones (Porter et al., 1996). Interestingly, a TSPO homolog has been predicted in some *Myxobacteria* species ^5^.

To date, no *Pseudomonas* species has been shown to produce such compounds, and the genes encoding the required enzymes are actually not predicted in the *Pseudomonas* database^1^. It may be conceivable that these bacteria could perceive or transport exogenous cholesterol or even structurally-related tetracyclic molecules like hopanoids for example (Fan et al., 2012). It is also possible that the functions of the Pseudomonas TSPO were evolutionary older, and that the possible involvement of mitochondrial TSPO in cholesterol transport and steroidogenesis occurred much later.

#### TSPO and iron homeostasis

**FIGURE 3:**
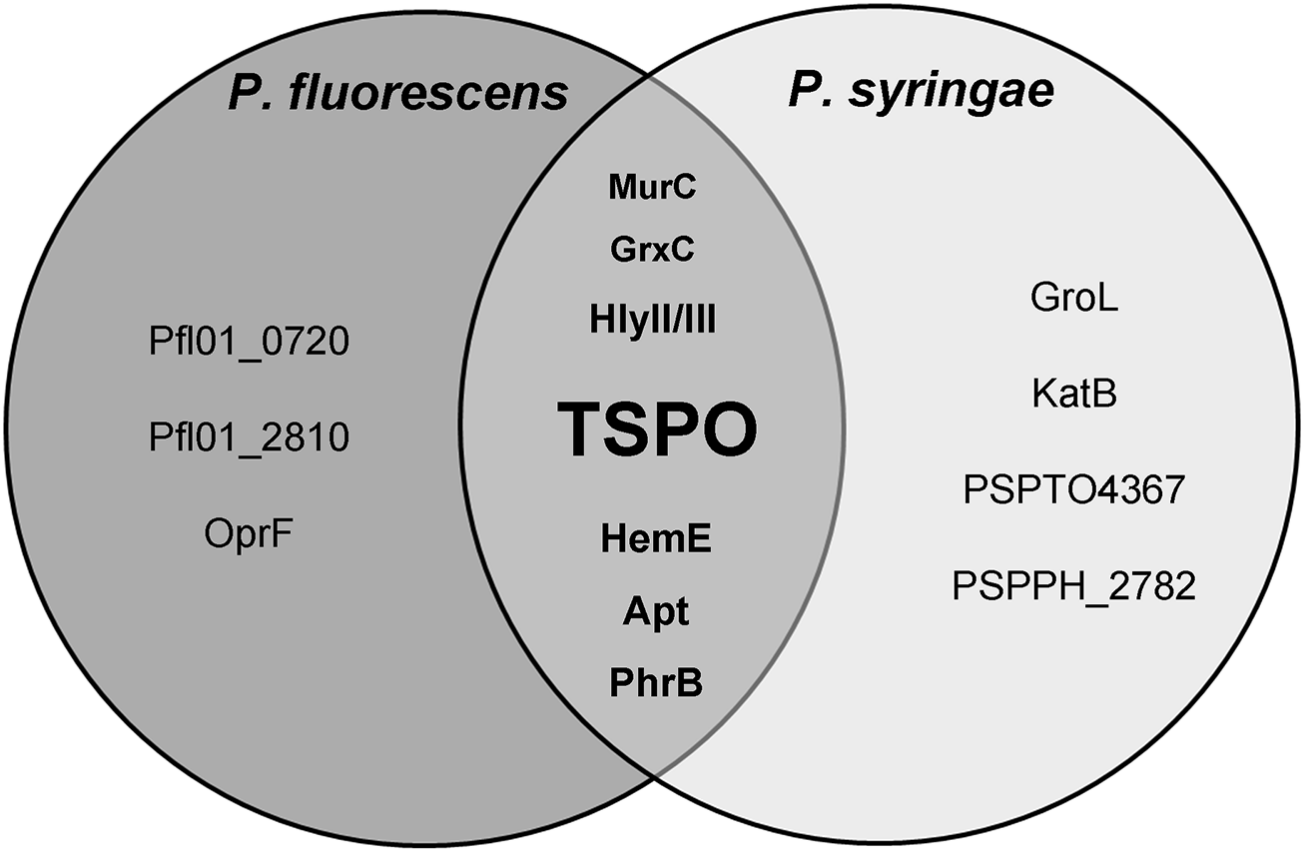
**Representation of the predicted interactions of TSPO in fluorescent *Pseudomonas*.** This Venn diagram was established using STRING version 9.05 software. Apt: adenine phosphoribosyl transferase; GroL, chaperone subunit; GrxC, monothiol glutaredoxin; HemE, uroporphyrinogen-III decarboxylase; HlyII/III, haemolysin II/III; KatB, catalase; MurC, UDP-*N*-acetylmuramate-L-alanine ligase; PhrB, type 1 deoxyribodipyrimidine photo-lyase; OprF, major outer membrane porin; Pfl01_0720, NAD-dependent epimerase/dehydratase; Pfl0-1_2810, PAS/PAC sensor HyHK histidine kinase; PSPTO4367, ortholog of the thiol oxydoreductase; PSPPH_2782, transposase.

To get further insights into the putative protein functions in *Pseudomonas*, TSPO’s functional interactions were retrieved from STRING database (von Mering et al., 2007), in *Pseudomonas syringae* (strains B728a, 1448A and DC3000) and *Pseudomonas fluorescens* (strains SBW25, Pfl0-1) since STRING data were not available for either *Pseudomonas fluorescens* A506 or *Pseudomonas poae* RE^∗^1-1-14. STRING is a database of known and predicted protein interactions, including physical and functional associations, which are derived from four sources including genomic context, high-throughput experiments, coexpression and literature. Only interactions with high confidence levels (>0.7) were kept. Common TSPO partners in each of the five studied strains were then represented as a Venn diagram (**Figure 3**; Chen and Boutros, 2011). This STRING-based study should be understood as a first hint where to search.

HemE is found to interact with TSPO in each of the studied strains, as well as in *R. sphaeroides* and *Sinorhizobium meliloti* (STRING), suggesting a putative robust link between the two proteins. HemE is an uroporphyrinogen-III decarboxylase that produces coproporphyrinogen III, also called apoferritin. Ferritin and ferritin-like molecules store Fe^3+^ as a mineral in their hollow cavities, effectively concentrating iron levels to orders of magnitude higher than those permitted by its low solubility (Yao et al., 2011). Iron is required by most organisms, but it is potentially toxic due to its low solubility, and to its tendency to potentiate the production of reactive oxygen species (ROS). The reactivity of iron is counteracted by bacteria using the same strategies employed by eukaryotes, namely by sequestering the metal into ferritin. These pivotal contributions made by ferritin-like molecules to iron homeostasis are manifested by their presence in all three domains of life with remarkable conservation of structure and function, despite very low sequence conservation (<20%; Grossman et al., 1992).

In addition to this major function in iron scavenging, apoferritin is also an intermediate of porphyrin and heme biosynthesis. Notably, in *R. sphaeroides*, TSPO has been shown to act as a negative regulator in the expression of *hemN* (Yeliseev and Kaplan, 1999), encoding a protein involved in heme trafficking. In mammals, coproporphyrinogen III is transported into the mitochondrial space where its conversion to PPIX takes place. PPIX is then converted into PPIX, which then sequester iron (Fe^2+^), thus forming heme (Kabe et al., 2006). The intra-mitochondrial accumulation of PPIX in response to iron starvation is toxic, and TSPO has been involved in mitochondrial detoxification, at least partly through PPIX export (Yeliseev and Kaplan, 1999; Mesenholler and Matthews, 2000). Remarkably, in the plant *A. thaliana*, in which heme biosynthesis is mainly localized in plastids (Mochizuki et al., 2010), TSPO has been proposed to participate in the interaction between the plastidal and the mitochondrial tetrapyrrole biosynthetic pathway, most likely by transporting the protoporphyrinogen from the plastids to the mitochondrial site of heme formation (Lindemann et al., 2004). Plant TSPO is also involved in scavenging unbound heme and porphyrins (Vanhee et al., 2011), and has recently been proposed to function as an autophagy receptor for toxic porphyrins leading to their specific degradation (Veljanovski and Batoko, 2014). Recently, bacterial TSPOs have been shown to catalyze rapid porphyrin degradation in a light- and oxygen-dependent manner – a reaction that is inhibited by the synthetic TSPO ligand, PK 11195 – and by mutations of conserved residues, which affect either porphyrin binding or catalytic activity (Ginter et al., 2013). Taken together these data suggest that TSPOs are ancient evolutionary conserved enzymes mediating porphyrin catabolism most likely with the consumption of ROS (Ginter et al., 2013).

#### TSPO and oxidative stress response

Iron is both an essential nutrient for the growth of microorganisms, as well as a dangerous metal due to its capacity to generate ROS via the Fenton reaction. For these reasons, bacteria must tightly control the uptake and storage of iron in a manner that restricts the build-up of ROS. Therefore, the control of iron homeostasis and responses to oxidative stress are intimately coordinated (Cornelis, 2010). The link between TSPO and the response to ROS is strengthened by the fact that several proteins that are involved in the oxidative stress response were predicted to interact with *Pseudomonas* TSPO. This was the case for example of the monothiol glutaredoxin GrxC that was found in fluorescent *Pseudomonas* TSPO interactome (**Figure 3**), which is involved in the bacterial oxidative stress response and in iron homeostasis (Cabiscol et al., 2000). In *E. coli*, GrxC is able to bind to glutathione, helping to maintain the redox status of the cell (Lillig et al., 2008). GrxC can then reduce the oxidized redox-response master regulator OxyR, thus creating a feedback loop that regulates OxyR activity (Zheng et al., 2001). In addition, GrxC is also a player in iron homeostasis, since it has been shown to bind Fe–S clusters, and it is also involved in some aspects of Fe–S cluster biogenesis and regulation (Iwema et al., 2009; Couturier et al., 2011; Boutigny et al., 2013). In the fungi, *Saccharomyces cerevisiae* and *Schizosaccharomyces pombe*, GrcX also plays essential roles in intracellular iron signaling, iron trafficking, and maturation of Fe–S cluster proteins (Mühlenhoff et al., 2010; Mapolelo et al., 2013). Common to each of the studied *Pseudomonas* TSPO functional networks is also PhrB (**Figure 3**), a conserved photolyase, the structure of which contains a [4Fe–4S] cluster as a cofactor bound to the catalytic domain (Zhang et al., 2013). Of note, PhrB was also predicted to interact with *R. sphaeroides* TSPO (STRING). Photolyases are enzymes involved in DNA repair and cell cycle regulation under stress conditions, which are commonly associated with the bacterial SOS response (Aravind et al., 2013). Interestingly, in mammals, mitochondria are the central cellular compartment for Fe–S cluster biogenesis, which is implicated in mitochondrial respiration and DNA repair (Stehling et al., 2014). Thanks to their chemical versatility, Fe–S centers act as catalysts or redox sensors (Stehling et al., 2014), and they are thought to rank among the most ancient and versatile inorganic cofactors found in all kingdoms of life (Beinert et al., 1997). The link between TSPO and ROS is further strengthened by the predicted interactions between *Pseudomonas syringae* TSPOs and both catalase KatB and orthologs of the thiol oxydoreductase PSPTO4367 (**Figure 3**), which are two key enzymes in almost all living organisms that are used to protect themselves against oxidative stress (Cabiscol et al., 2000; Mishra and Imlay, 2012). Interestingly, KatB was also linked to TSPO in *Sinorhizobium meliloti* (STRING data not shown).

In mammals, the mitochondrial location of the TSPO is interesting as it is well known that mitochondria are a main source of cellular ROS (Lenaz, 1998). It has been shown that oxidative stress modulates TSPO structure and function (Delavoie et al., 2003). Increased ROS levels lead indeed to TSPO polymerization through di-tyrosine formation, which modulates the function of TSPO in cholesterol transport, since polymer formation induced by ROS increased both TSPO ligand binding and cholesterol-binding capacities (Delavoie et al., 2003). Vice versa, TSPO appears to be an essential participant in ROS generation at mitochondrial levels (Veenman et al., 2007; Zeno et al., 2009; Choi et al., 2011). In the liver, TSPO was found in co-localization with a ROS scavenger, the mitochondrial manganese-dependent superoxide dismutase (SOD; Fischer et al., 2001). In the moss plant *Physcomitrella patens* (Pp), Pp*tspo1* knockout mutants were shown to be impaired in their mitochondrial PPIX uptake and produced elevated levels of intracellular ROS, implying a role of PpTSPO1 in redox homeostasis (Frank et al., 2007; Vanhee et al., 2011). TSPO-deficient plants were shown to be under permanent oxidative stress and suffer from disturbed redox homeostasis (Lehtonen et al., 2012). Abiotic stresses, among which salt, drought or cold, transiently up-regulate heme biosynthesis that is required for the activity of ROS scavengers, and simultaneously induce the expression of TSPO (Frank et al., 2007). In *A. thaliana*, AtTSPO is expressed in dry seeds and can be induced in vegetative tissues by osmotic and salt stresses or by abscisic acid (ABA) treatments, suggesting that AtTSPO is specifically induced by water-related stress (Guillaumot et al., 2009a; Balsemão-Pires et al., 2011). Interestingly, the stress phytohormone ABA regulates plant water status through regulation of stomatal closure (Finkelstein et al., 2002; Nambara and Marion-Poll, 2005). The increase in active ABA levels in plant cells during water-related stress regulates the expression of ABA-responsive genes, among which *tspo* (Guillaumot et al., 2009a,b; Balsemão-Pires et al., 2011).

Taken together, these data suggest that *Pseudomonas* TSPO belongs to an evolutionarily conserved coordinated network that is involved in controlling iron and redox homeostasis.

#### Virulence

TSPO was predicted to interact with hemolysin II/III (**Figure 3**, HlyII/III). These proteins are exoenzymes that exhibit cytolytic activity against eukaryotic cells, which are involved in retrieving iron from eukaryotic heme (Guillemet et al., 2013). Such enzymes belong to the virulence factor arsenal of pathogenic bacteria, which enable bacterial survival among hosts in which the iron concentration is limited (Cornelis, 2010). The link between TSPO and virulence is also supported by a recent transcriptomic study in which *tspo* expression was increased in *Pseudomonas syringae sp. phaseolicola* NPS3121 cultured at a low (18∘C) rather than at an optimal (28∘C) growth temperature (Arvizu-Gomez et al., 2013). Interestingly, low temperature is the cue for pathogens to produce virulence factors, including toxins, cell wall-degrading enzymes, and effector proteins (Picot et al., 2004; Arvizu-Gomez et al., 2013), suggesting that TSPO may be part of a virulence-related network. It is interesting to note that in *Pseudomonas syringae pv. syringae* B728a and in *Pseudomonas syringae pv. tomato* DC3000, *tspo* is located in the vicinity near genes that encode homologs of virulence factors, SrfB and SrfC, of *Salmonella enterica* (Chapalain et al., 2009). These data are also in agreement with those showing that TSPO should be related to the expression of the cytotoxic activity of *Pseudomonas fluorescens* (Chapalain et al., 2009). Interestingly, PK 11195 was shown to increase adhesion and biofilm formation activities while decreasing the cytotoxic (apoptotic) effect of *Pseudomonas fluorescens* MF37 (Chapalain et al., 2009). These observations are consistent with the fact that the growth of *Pseudomonas* in biofilms or aggregates is associated with a decrease in virulence (Staudinger et al., 2014), and this supports the hypothesis that, as in *Pseudomonas syringae*, TSPO is involved in virulence expression.

In the three studied *Pseudomonas syringae* strains, the STRING analysis predicted that TSPO interacts with the chaperone subunit, GroL (**Figure 3**), a protein that belongs to the heat shock sigma factor RpoH regulon. Since RpoH is involved in the response to stresses leading to misfolded or aggregated proteins, it suggests that TSPO may be linked to this type of general stress response. Interestingly, such functions have been proposed in plant TSPOs (Balsemão-Pires et al., 2011). Furthermore, in addition to their well-documented function as molecular chaperones, GroEL proteins are increasingly recognized as exhibiting surprising additional so-called “moonlighting” functions (Henderson et al., 2013). Perhaps the most astonishing feature of members of the GroEL family is that they can demonstrate toxic activities against eukaryotes (Kupper et al., 2014).

#### Membrane and cell wall biogenesis and/or integrity

TSPO was also predicted to interact with three proteins involved in membrane and cell wall biogenesis, including a putative nucleoside-diphosphate-sugar epimerase Pfl01_0720, the UDP-*N*-acetylmuramate-alanine ligase MurC, and OprF (**Figure 3**). OprF is the major OM porin of members belonging to the *Pseudomonas* genus, which is homologous to the mitochondrial VDAC. Functional links between OprF and TSPO have been demonstrated in *Pseudomonas fluorescens* MF37 (Chapalain et al., 2009). Notably, in addition to its structural role in anchoring the OM to the peptidoglycane layer, OprF has been shown to be required for full virulence expression in the human pathogen *Pseudomonas aeruginosa* (Fito-Boncompte et al., 2011). OprF is also a receptor for human IFNγ, allowing the bacteria to sense the host’s immune state (Wu et al., 2005). Since all of the *Pseudomonas* are not pathogens, it has further been proposed that this protein could act as a more general environmental sensor (Wagner et al., 2006). The interaction between TSPO and OprF suggests that they could participate in a common sensing/transducing network. This hypothesis is also supported by the observation of a possible functional link between TSPO and the adenine phosphoribosyl transferase (Apt, **Figure 3**), which enables the transfer of a phosphoribosyl group on adenine, thus producing AMP (Becerra and Lazcano, 1998). Interestingly, in mammals, TSPO is possibly associated with an ANT, which is localized in the inner membrane of mitochondria, and it plays an essential role in transporting ADP into the mitochondrial matrix and ATP out from the matrix for cell utilization (Leung et al., 2013). As such, we cannot exclude that as in eukaryotes, bacterial TSPO should form a ternary complex with an adenine-associated substrate translocating protein.

#### Signaling and signal transduction

**FIGURE 4:**
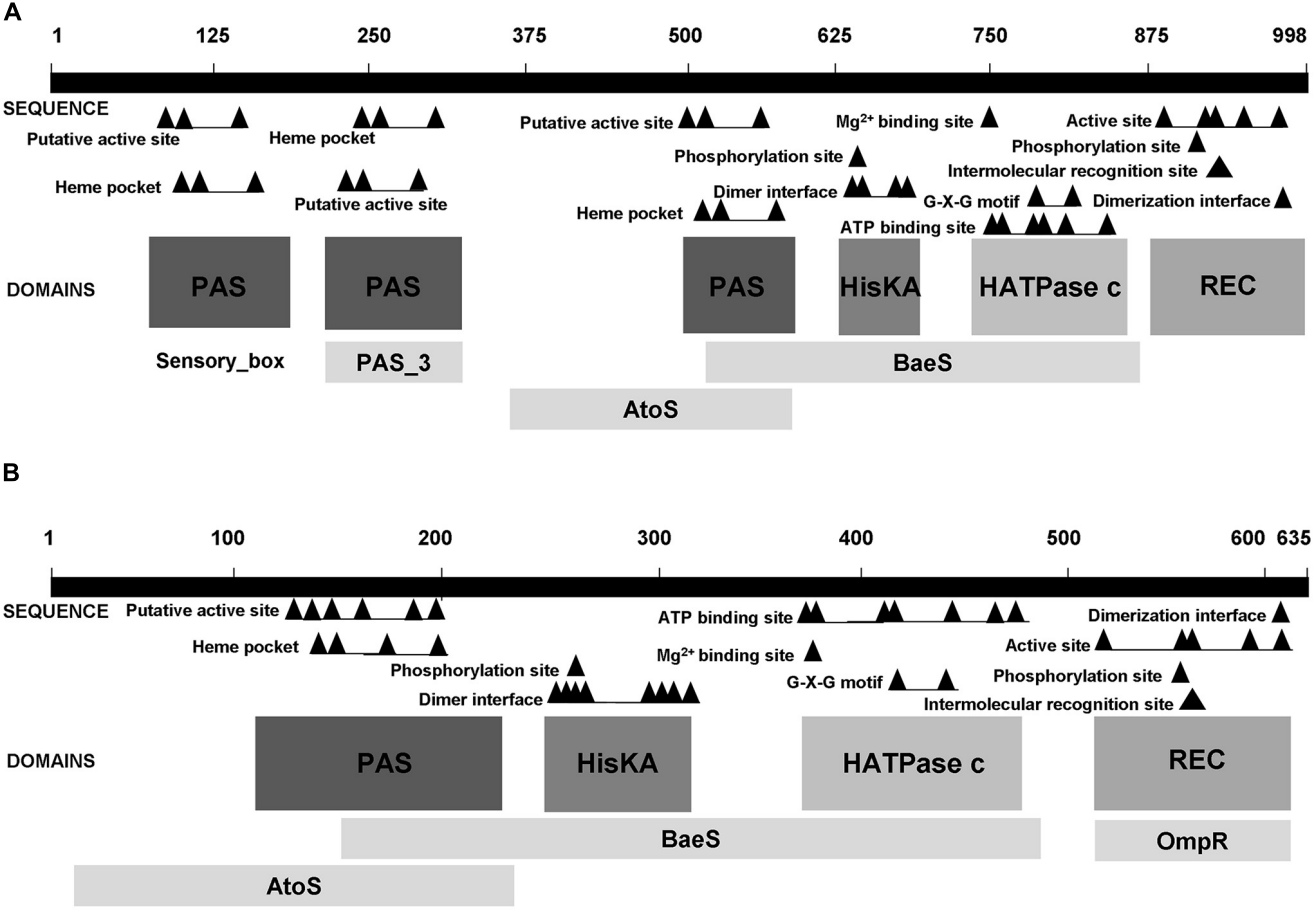
**Comparison of the hybrid histidine kinases HyHK (Pfl01_2810) in *Pseudomonas fluorescens* Pf0-1 **(A)** and StoS in *Xanthomonas oryzae pv. Oryzae***(B)**.** HATPase-c, catalytic and ATP-binding domain; HisKa, dimerization and phosphotransfer domain; PAS, sensor domain; REC, response-regulator domain.

As mentioned in Section “Functions”, TSPO has been shown to be involved in gene regulation, noticeably in *R. sphaeroides*, in which TSPO has been proposed as an oxygen sensor (Yeliseev et al., 1997), and in *Sinorhizobium melilotti* (Davey and de Bruijn, 2000). In the latter, TSPO is involved in regulating the expression of the nutrient deprivation induced (*ndi*) locus. In this bacterium, the two component sensor FixL, has been shown to be required for full induction of the *ndi* locus expression. Interestingly, these authors demonstrate that TspO appears to be epistatic to the histidine kinase (HK) FixL, since no expression is observed in the *tspO* mutant, even though FixL is still present (Davey and de Bruijn, 2000). Using the STRING algorithm to investigate the putative interactions of TSPO in *Pseudomonas fluorescens* Pf0-1, we observed a potential link between TSPO and a hybrid HK (HyHK, Pfl0-1_2810; **Figure 3**). Moreover, in this bacterium, the two genes appear to be located in the same operonic structure^1^, suggesting the presence of genetic and functional links. Two component signal transduction systems generally consist of a sensor HK and a response regulator (RR) that contains a receiver or response regulator domain (REC). Hybrid-type HKs (HyHKs) comprise a HK with a receiver domain within one molecule (Stock et al., 2000). Interestingly, the *Pseudomonas fluorescens* Pf0-1 HyHK is predicted to possess six functional domains, among which three PAS domains (**Figure 4**). In the bacterial kingdom, PAS domains are commonly positioned at the amino terminus of signaling proteins such as sensor HKs, and tandem and multiple PAS domains are common in individual proteins: about one third of PAS proteins contain two or more PAS domains (Henry and Crosson, 2011). Interestingly, PAS ligand binding either functions as a primary cue to initiate a cellular signaling response or provides the domain with the capacity to respond to secondary physical or chemical signals such as gas molecules, redox potential, or photons (Henry and Crosson, 2011). Noticeably, many PAS-domain proteins detect their signal by way of an associated cofactor such as heme (Henry and Crosson, 2011), and this may be the case for the *Pseudomonas fluorescens* Pf0-1 HyHK since a heme pocket has been predicted in each of the three PAS domains (**Figure 4**). Interestingly, the *Pseudomonas fluorescens* Pf0-1 HyHK shows some domain similarities with the rice pathogen *Xanthomonas oryzae pv. Oryzae*, HyHK StoS (i.e., the four C-terminal domains), as it includes a PAS domain with a heme pocket involving StoS as an oxygen sensor, a HisKa dimerization and phosphotransfer domain with an Mg^2+^ binding site, a HATPase energizing domain, and a receiver REC domain (**Figure 4**). Interestingly, StoS is activated by sensing low-oxygen concentrations, and it is involved in stress tolerance and virulence (Ikawa et al., 2014). As shown on **Figure 4**, the *Pseudomonas fluorescens* Pf0-1 HyHK, -as well as StoS-, shows domain homology with the BaeS HK, and to a lesser extend to the AtoS HK (**Figure 4**). In *E. coli*, the two component system (TCS) AtoSC has been involved in modulating diverse fundamental cellular processes such as short-chain fatty acid catabolism, poly-(R)-3-hydroxybutyrate biosynthesis and chemotaxis (Kyriakidis and Tiligada, 2009). In *E. coli*, the BaeSR TCS is involved in response to severe envelope stress like spheroplasting (Raffa and Raivio, 2002). Recently, it has been shown that, in *Salmonella enterica* serovar *Typhimurium*, BaeSR is required for expression of *sodA* and possibly of *sodB*, encoding two major SODs that are involved in ROS detoxification (Guerrero et al., 2013).

### THE CASE OF *Pseudomonas aeruginosa*

Dealing with *Pseudomonas* without mentioning its best known representative species, *Pseudomonas aeruginosa*, should appear incomplete, but its absence all throughout this review was only motivated by the lack of the *tspo* analog in the genome of *Pseudomonas aeruginosa*. Moreover, all attempts to identify even a truncated or partly deleted *tspo* sequence in *Pseudomonas aeruginosa* sequenced genomes were failures (Chapalain et al., 2009; Fan et al., 2012). This observation could appear as a simple illustration of the highly variable expression of bacterial TSPO. However, the situation should be more complex. Indeed, it has been shown that benzodiazepines improve the recovery of animals in the *Pseudomonas aeruginosa*-infected burned mouse model (Dugan et al., 2010), and a detailed analysis of the data indicates that the effect of benzodiazepines on the healing process is not due to direct stimulation of cutaneous cicatrization (Egydio et al., 2012). It is likely that there is no direct effect of benzodiazepines on *Pseudomonas aeruginosa* but the effects seen is due ot the modulatory effect of benzodiazepines on the immune system (Egydio et al., 2012). This is similar to the case of epinephrine, known since 1930 to have a major impact on bacterial infections but whose direct effect on bacteria was only recognized by the end of the 20th century (Lyte and Ernst, 1992) – well before its bacterial sensor was identified (Clarke et al., 2006; Hughes and Sperandio, 2008). In fact, although *Pseudomonas aeruginosa* does not express TSPO, its large genome encodes many sensors (receptors) and transporters and different porins, including OprF. In addition, other studies indicated that benzodiazepine analogs can exhibit *in vitro* direct antibacterial activities on *Pseudomonas aeruginosa* (Sharma et al., 2013), suggesting that *Pseudomonas aeruginosa* can sense benzodiazepines. Considering these findings, we cannot exclude the possibility that a sensor protein showing functional homologies with TSPO is expressed in *Pseudomonas aeruginosa*.

Interestingly, the budding yeast *Saccharomyces cerevisiae* is known to lack TSPO, which may be due to the loss of almost 90% of duplicated genes in this organism (Kellis et al., 2004). This characteristic makes *Saccharomyces cerevisiae* well-suited for heteroexpression studies and an ideal model system for studying the biochemical and pharmacological properties of TSPO (Riond et al., 1991). However, the lack of TSPO in *Saccharomyces. cerevisiae* also shows that TSPO is not essential for yeast viability and therefore other pathways could perform the functions attributed to the TSPO in other organisms.

## CONCLUSION

TSPO is a protein whose typical five trans-membrane helix structure has been remarkably preserved along the evolutionary process, but whose localization and functions evolved from α- and γ-proteobacteria to eukaryotes. As a membrane protein, TSPO may be involved in redox and iron homeostasis, and virulence expression as its regulatory networks could be intimately intermingled. As is the case with mitochondria, in *Pseudomonas*, bacterial TSPO could also take part in signal transduction and in establishing membrane integrity. As a result, TSPO appears to be a vital protein in fluorescent *Pseudomonas*.

## AUTHOR CONTRIBUTIONS

Charlène Leneveu-Jenvrin, Nathalie Connil, Emeline Bouffartigues, Vassilios Papadopoulos, Marc G. J. Feuilloley, Sylvie Chevalier wrote the manuscript and performed the *in silico* analysis.

## Conflict of Interest Statement

The authors declare that the research was conducted in the absence of any commercial or financial relationships that could be construed as a potential conflict of interest.

## Footnotes

1 http://www.pseudomonas.com

2 http://www.ncbi.nlm.nih.gov/gene/3559346

3 http://multalin.toulouse.inra.fr/multalin/multalin.html

4 http://www.pasteur.fr/

5 http://www.uniprot.org/
